# White Decay

**Published:** 1883-09

**Authors:** 


### WHITE DECAY.

A well-known dentist called the attention of a reporter to the effects of Allegheny River water on the teeth of a large portion of our citizens. He stated that there were more persons afflicted with white decay or crumbling teeth in this vicinity than in any other city in which he had practiced. The teeth of those afflicted with this form of disease were generally very white and they gradually crumbled into powder. He attributed the great prevalence of white decay here to the absence of lime in the drinking water. People suffered from acidity of the system, and lime was the alkali which would benefit them. In the eastern portion of Pennsylvania, or rather in countries where the people drank "hard" water, they generally had hard and sound teeth, but in communities where "soft" water was used the opposite result was found. He advised the drinking of lime water by people troubled with white decay.—*Pittsburgh Commercial Gazette.*

AFTER a boy is tired of hoeing potatoes, nothing seem to rest him more than to dig over a few square rods of greensward in search of bait.

Do not build on heaps of rubbish, fillings in with cess-pool refuse, chemical waste, or on swampy ground which cannot be drained. Thousands of houses have been so placed, and are now being so placed in suburbs of our towns.

AN old lady in Georgia, having lost all her patience, has sued a neighbor for eight dollars for coffee borrowed a cupful at a time. It is in order, to suggest that she has ample grounds for the suit.—*Lowell Courier.*

A GOOD WHITEWASH THAT WILL NOT RUB OFF.—Mix up half a pailful of lime and water ready to put on the wall; then take ¼ pint of flour, mix it up with the water, then pour on it boiling water in sufficient quantity to thicken it; pour it while hot into the whitewash, stir together and it is ready for use.

